# 2181. Avibactam Prevents the *in vitro* Development of Imipenem Resistance in non-Carbapenemase-Producing *Klebsiella pneumoniae*

**DOI:** 10.1093/ofid/ofad500.1803

**Published:** 2023-11-27

**Authors:** Ana M Quesille-Villalobos, Consuelo Bolivar, Juan José Nicolai, Veronica Herrera, Jose R W Martinez, Valeria Quiroz, Lorena Dias, Manuel Alcalde-Rico, Araos Rafael, Patricia Garcia, José M Munita

**Affiliations:** Universidad del Desarrollo, Santiago, Region Metropolitana, Chile; Universidad Andres Bello, Santiago, Region Metropolitana, Chile; Universidad del Desarrollo, Santiago, Region Metropolitana, Chile; Universidad del Desarrollo, Santiago, Region Metropolitana, Chile; Universidad del Desarrollo, Santiago, Region Metropolitana, Chile; Universidad del Desarrollo, Santiago, Region Metropolitana, Chile; Univerdidad del Desarrollo, Santiago, Region Metropolitana, Chile; Hospital Universitario Virgen Macarena, Santiago, Region Metropolitana, Chile; Universidad del Desarrollo, Santiago, Region Metropolitana, Chile; Pontificia Universidad Catolica de Chile, Santiago, Region Metropolitana, Chile; Clínica Alemana - Universidad del Desarrollo, Santiago, Chile

## Abstract

**Background:**

Carbapenem resistance (CR) in *Klebsiella pneumoniae* is most frequently mediated by the production of carbapenemases. However, non-carbapenemase-producing *K. pneumoniae* (non-CP-*Kpn*) have been well documented. Overexpression of narrow β-lactamases can play an important role in the development of CR in the absence of carbapenemases. Here, we investigated the role of avibactam, a potent β-lactamase inhibitor, in preventing the *in vitro* development of CR in a non-CP-*Kpn*.

**Methods:**

We used SCL-1, a non-CP-*Kpn* strain recovered from the bloodstream of a patient that developed CR *in vivo* during treatment with imipenem (IMI). SCL-1 was exposed to serial passages of increasing concentrations of IMI alone [0.25-16 μg/mL] or in combination with avibactam (IMI-AVI) during 8 days. In the case of IMI-AVI, the concentration of AVI was fixed at 4 μg/mL. Evolution assays were performed in triplicate and considered 3 independent evolutionary lines (ELs). MICs at each time-point were measured to IMI and IMI-AVI by broth microdilution as per CLSI. Growth curves of SCL-1 and evolved strains were performed to assess fitness. The expression levels of narrow spectrum β-lactamases *bla*_OXA-1_,*bla*_OXA-10_, and of the ESBL *bla*_CTX-M-15_ were measured by RT-qPCR.

**Results:**

*In vitro* exposure to IMI led to CR, with the MICs increasing from 0.5 μg/mL to 8 μg/mL in all 3 ELs. In contrast, all ELs exposed to IMI-AVI remained fully susceptible to IMI after 8 days, with a final MIC of 1 μg/mL. In addition, the MIC to IMI of the evolved, IMI-resistant strains, in the presence of AVI (4 μg/mL) dropped from 8 to 1 μg/mL. Growth curves revealed that IMI-resistant strains evolved in IMI alone exhibited a growth defect as compared to both SCL-1 and the susceptible strains evolved in IMI-AVI. Finally, compared to the SCL-1, the expression level of *bla*_OXA-1_ was significantly increased (*p*< 0.05) in all evolved isolates that developed IMI resistance (MIC=8 μg/mL). No significant changes were observed in the expression of *bla*_OXA-10_ and *bla*_CTX-M-15_ (Figure 1).

**Figure 1.**
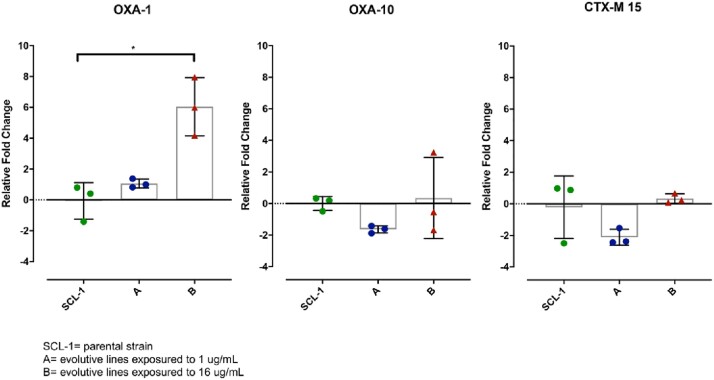
RT-qPCR analysis of β-lactamase gene transcript levels for SCL-1 and evolutive lines (A and B). Data shown are individual data points with mean±SD superimposed.

**Conclusion:**

Our findings suggest that the addition of AVI can prevent the *in vitro* development of IMI resistance in non-CP-*Kpn*. Our results have important implications for the clinical use of AVI as a strategy to combat CR in non-CP-*Kpn* infections.

**Disclosures:**

**All Authors**: No reported disclosures

